# Sodium ferulate and *n*-butylidenephthalate combined with bone marrow stromal cells (BMSCs) improve the therapeutic effects of angiogenesis and neurogenesis after rat focal cerebral ischemia

**DOI:** 10.1186/s12967-016-0979-5

**Published:** 2016-07-28

**Authors:** Qian Zhang, Yonghua Zhao, Youhua Xu, Zhenwei Chen, Naiwei Liu, Chienchih Ke, Bowen Liu, Weikang Wu

**Affiliations:** 1 State Key Laboratory of Quality Research in Chinese Medicine, Faculty of Chinese Medicine, Macau University of Science and Technology, Avenida Wai Long, Taipa, Macao, People’s Republic of China; 2 Biomedical Imaging Research Center, National Yang Ming University, Taipei, Taiwan; 3 Department of pathophysiology, Zhongshan School of Medicine, Sun Yat-sen University, Guangzhou, People’s Republic of China

**Keywords:** Angiogenesis, Bone marrow stromal cells, Ischemic stroke, *n*-Butylidenephthalide, Neurogenesis, Sodium ferulate

## Abstract

**Background:**

Studies have indicated that bone marrow stromal cell (BMSC) administration is a promising approach for stroke treatment. For our study, we chose sodium ferulate (SF) and *n*-butylidenephthalide (BP) combined with BMSC, and observed if the combination treatment possessed more significant effects on angiogenesis and neurogenesis post-stroke.

**Methods:**

We established rat permanent middle cerebral artery occlusion (MCAo) model and evaluated ischemic volumes of MCAo, BMSC, SF + BP, Simvastatin + BMSC and SF + BP + BMSC groups with TTC staining on the 7th day after ischemia. Immunofluorescence staining of vascular endothelial growth factor (VEGF) and brain derived neurotrophic factor (BDNF), as well as immunohistochemistry staining of von Willebrand factor (vWF) and neuronal class III β-tubulin (Tuj1) were performed in ischemic boundary zone (IBZ), furthermore, to understand the mechanism, western blot was used to investigate AKT/mammalian target of rapamycin (mTOR) signal pathway in ischemic cortex. We also tested BMSC derived-VEGF and BDNF expressions by western blot assay in vitro.

**Results:**

SF + BP + BMSC group obviously decreased infarction zone, and elevated the expression of VEGF and the density and perimeter of vWF-vessels as same as Simvastatin + BMSC administration; moreover, its effects on BDNF and Tuj1 expressions were superior to Simvastatin + BMSC treatment in IBZ. Meanwhile, it showed that SF and BP combined with BMSC treatment notably up-regulated AKT/mTOR signal pathway compared with SF + BP group and BMSC alone post-stroke. Western blot results showed that SF and BP treatment could promote BMSCs to synthesize VEGF and BDNF in vitro.

**Conclusions:**

We firstly demonstrate that SF and BP combined with BMSC can significantly improve angiogenesis and neurogenesis in IBZ following stroke. The therapeutic effects are associated with the enhancement of VEGF and BDNF expressions via activation of AKT/mTOR signal pathway. Furthermore, triggering BMSC paracrine function of SF and BP might contribute to amplifying the synergic effects of the combination treatment.

**Electronic supplementary material:**

The online version of this article (doi:10.1186/s12967-016-0979-5) contains supplementary material, which is available to authorized users.

## Background

Bone marrow stromal cells (BMSCs) have been recognized as a distinguished candidate for ischemic stroke treatment in terms of their multi-potentiality [1]. Numerous studies suggested BMSC administration could enhance endogenous neurogenesis and angiogenesis; moreover, part of them showed neuronal and endothelial morphologies in the ischemic boundary zone (IBZ) post-stroke [2–6]. Because neurogenesis would directly replace dead neurons and angiogenesis would result in new blood vessel formation, which might obviously increase oxygen, glucose, nutrients supplement and neural stem cells (NSCs) migration to IBZ, evidence suggested that neurogenesis and angiogenesis played a central role in the neurological functional recovery after stroke [7]. A recent research demonstrated neurogenesis was coupled with angiogenesis, and neurorestorative therapy should focus more on the interaction between them [8].

Reports indicate the combination treatment with BMSC and pharmacological agent is superior to any single treatment method. For example, Simvastatin treatment had been found to enhance neurogenesis and angiogenesis after stroke [9], and BMSC combined with Simvastatin could significantly facilitate BMSCs differentiation into endothelial cells and improve arteriogenesis and angiogenesis, which was favorable to ameliorate functional outcome after cerebral ischemia [10–12].

Studies found that ferulic acid (FA) could improve neurological function and decrease infarction size in middle cerebral artery occlusion (MCAo) rats through anti-oxidative and anti-inflammatory effects, and it could also advance angiogenesis via increasing vascular endothelial growth factor (VEGF), platelet-derived growth factor and hypoxic-induced factor (HIF)-1α expressions in human umbilical vein endothelial cells [13, 14]. Our previous studies also suggested that sodium ferulate (SF, sodium salt of FA) combined with BMSC administration further decreased rat brain ischemic injury and increased neurogenesis at the 3rd day after ischemic stroke [16, 17]. As an alkylphthalide, *n*-butylidenephthalide (BP) could inhibit lipopolysaccharide-induced productions of nitric oxide, tumor necrosis factor-α and interleukin-1β from rat brain reactive microglia, thus contributing to the homeostasis of inflammatory mediation [15]. BP also had the capacities of keeping embryonic stem cell pluripotency by activating Jak2/Stat3 pathway and enhancing induced pluripotent stem cell (iPSc) generation efficiency [18]. SF and BP are active components from Radix Angelica Sinensis, and both of them suggest beneficial effects on stroke pathological processes. To investigate if SF and BP combined with BMSC could further advance angiogenesis and neurogenesis in rat ischemic stroke, we designed the present study; meanwhile, the potential mechanism was explored.

## Methods

### Therapeutic agents and reagents

SF and BP were obtained from Shanghai Institute of Materia Medica, Chinese Academy of Sciences. GIBCO^®^ Dulbecco’s modified Eagle medium/F12 (DMEM/F12) was product of Life Technologies (USA). Primary antibodies for CD90-PE, CD45-FITC, CD34-PerCP were from Santa Cruz (California, USA). 2,3,5-triphenyltetrazolium chloride (TTC), MTT and Simvastatin were purchased from Sigma-Aldrich (St. Louis, MO, USA). Primary antibodies of VEGF, von Willebrand factor (vWF), brain derived neurotrophic factor (BDNF) and neuronal class III β-tubulin (Tuj1) were products of Abcam (Cambridge, UK). Avidin–biotin peroxidase complex (ABC) kit and 3,3′-diamino-benzidine (DAB) kit were from Zymed Laboratories Inc (California, USA). Primary antibodies of AKT, p-Akt, mTOR and p-mTOR were obtained from Cell Signaling Technology, Inc. (Danvers, USA). The other materials and reagents were from commercial sources.

### Primary culture of BMSC and identification

BMSCs were obtained and cultured as our previous report [16]. Briefly, the cells were harvested aseptically from bone marrow of the tibias and femurs in 50–60 g male Sprague–Dawley (SD) rats and cultured with DMEM/F12 cell medium supplemented with 10 % fetal bovine serum and 1 % penicillin–streptomycin in a cell culture flask at 37 °C and 5 % CO_2_. New culture medium was replaced once every 48 h. The cells were digested and passaged with 0.25 % trypsin (HyClone) during cell logarithm growth period with the cell fusion of 80 %.

To evaluate the BMSC purity, flow cytometry (Becton, Dickinson and Company, USA) was performed to identify CD90, CD45 and CD34 surface markers of cultured cells. The cells from passage 3 were collected and fixed with 4 % paraformaldehyde for 5 min and washed with 0.1 M phosphate-buffered saline (PBS). After that, they were adjusted to the density of 1 × 10^7^/ml and were respectively incubated with fluorescence-conjugated antibodies including CD90-PE, CD45-FITC, CD34-PerCP and PBS (negative control) in a black chamber at 4 °C for 30 min. After washing with PBS, the cells were analyzed by a flow cytometer equipped with the Cellquest system (Becton, Dickinson and Company, USA).

### The establishment of permanent middle cerebral artery occlusion model and experimental group definition

The study conforms to the National Institute of Health Guide for the Care and Use of Laboratory Animals (NIH Publications No. 80-23) revised in 1996 for scientific purposes. All experimental procedures were approved by the Institutional Animal Care and Use Committee of Macau University of Science and Technology. Adult male SD rats (Guangdong Medical Laboratory Animal Center, Foshan, Guangdong, China) weighing 240–260 g were used in all experiments. Permanent middle cerebral artery occlusion (pMCAo) was established according to our previous method [16]. In brief, the rats were anaesthetized with 10 % (w/v) chloral hydrate (3.0 ml/kg, i.p.) and their body temperature was maintained at 40 °C by animal heating pad. 4–0 surgical nylon suture (length of 20–22 mm determined by body weight) coated with polylysine was inserted into the lumen of the right common carotid and advanced into the internal carotid artery until it obstructed the origin of the middle cerebral artery. Finally, the neurological function was evaluated using a 5-point scale neurological deficit score as reported by Longa and colleagues [19]. Only animals with the score of 2–3 were selected for the subsequent studies (0 = no deficit, 1 = failure to extend left paw, 2 = circling to the left, 3 = falling to the left, 4 = unable to walk spontaneously and consciousness). Sham operated animals underwent the same surgery but without nylon suture inserted.

One hundred and twenty MCAo rats were randomly divided into 5 groups (24 rats per group), including MCAo group, BMSC group, Simvastatin (Sim) + BMSC group (as positive control), SF + BP group and SF + BP + BMSC group. One millilitre of PBS or BMSC suspension solution (2 × 10^6^ cells/ml) was intravenously injected into MCAo, SF + BP or BMSC alone, Sim + BMSC and SF + BP + BMSC groups via caudal vein at 3 h after operation; and 1 ml of PBS or SF (60 mg/kg) was intraperitoneally injected into MCAo, BMSC, Sim + BMSC group or SF + BP and SF + BP + BMSC groups once a day for continuous 7 days, the dosage SF were determined according to our previous report [17]; Simvastatin (1 mg/kg) [10] was gavaged daily for 7 days in Sim + BMSC group and BP (10 mg/kg) was subcutaneously injected into SF + BP and SF + BP + BMSC groups once a day for continuous 3 days respectively, the ratio of SF and BP dosage was defined according to preliminary experimental result (see Additional file 1).

### TTC staining

To observe the volume of infarction zone in rat brain on the 7th day after treatment, TTC staining was applied. Rats (n = 5) in each group were deeply anaesthetized with 10 % (w/v) chloral hydrate, and the brains were removed quickly and placed at −20 °C for 15 min, sections of 2.0 mm thickness were cut and stained with 2 % solution of TTC at 37 °C for 30 min. The cross-sectional area of each slice in infarcted brain was measured using image analysis software (Image-Pro Plus Version 6.0, USA). The infarction volume was calculated by the corrected infarct volume (CIV). The formula: CIV (%) = [contralateral hemisphere volume−(ipsilateral hemisphere volume−infarct volume)]/contralateral hemisphere volume × 100 [20].

### Immunofluorescence staining

On the 7th day after ischemic stroke, rats (n = 6) in each group were anaesthetized with 10 % chloral hydrate. Brains were fixed by transcardial perfusion with saline, followed by perfusion and immersion in 4 % paraformaldehyde. Fresh frozen sections of 5 μm thickness were cut on a cryostat microtome (Thermo fisher Scientific Shandon Cryotome FSE). Every 5th coronal section for a total of 6 sections was used for immunofluorescence staining. Prior to all staining procedures, tissue slices were extensively rinsed with 0.02 M PBS and blocked with 5 % normal goat serum in 0.02 M PBS containing 0.3 % Triton X-100 for 1 h. To identify the expressions of VEGF and BDNF in the IBZ, the slices incubated with rabbit primary antibodies to VEGF (1:50) and BDNF (1:200) at 4 °C overnight, followed by secondary antibodies to Alexa Fluor 488 labeled goat anti-rabbit IgG (H + L) (Life Technologies, USA) at 37 °C for 1 h. Nuclei were finally stained with 4′,6-diamidino-2-phenylindole in all images. The slices were visualized and digitally photographed with a confocal laser scanning microscope (Zeiss LSM710, Germany), 5 non-overlapping fields of one slice were randomly observed under a magnification of 10 × 40 in confocal images. To quantitatively analyze the proteins expression, Image-Pro Plus software was applied.

### Histological and immunohistochemical staining

Rat brains (n = 6) were fixed with 4 % paraformaldehyde, and embedded in paraffin and cut into a series of 6 μm thick sections. For a morphological analysis of vessels and newborn neurons, samples were rinsed with Dulbecco’s phosphate buffered saline (Sigma, USA) containing 0.01 % Tween-20 and immersed in 3 % H_2_O_2_/methanol for 15 min to inhibit endogenous peroxidase activity. Subsequently, brain sections were incubated with 10 % normal goat serum for 20 min at room temperature, and then incubated with primary antibodies against vWF (1:100) and Tuj1 (1:200) at 4 °C for overnight. Following incubation with secondary antibody and ABC kit, sections were colored with DAB kit, and then stained with hematoxylin as a counterstain. Five slides were taken from each brain and each slide was randomly chose 5 non-overlapping fields to observe the expressions of vWF and Tuj1 under BX51 microscopy (Olympus, Japan). The determinant of vascular density was assessed by the number of positive vWF-vessels/mm^2^ using the Aperio ImageScope 12.3 (Leica), simultaneously vWF positive vascular perimeter and Tuj1 expression were quantified using Image-Pro Plus software in IBZ.

### MTT assay and experimental group divided in vitro

In order to find the optimal dosage of SF and BP treatment for BMSC, the cells (1 × 10^5^ cells/ml) were seeded in 96-well plates and incubated with different concentrations of SF (400, 200, 100, 50, 25, 5, 1, 0.1 and 0.01 μg/ml), BP (4, 2, 1, 0.75, 0.5, 0.25, 0.125, 0.01 and 0.001 μg/ml) for 48 h. According to the results, we found the SF concentration of 1 μg/ml and the BP concentration of 0.75 μg/ml had significant capacities of enhancing the viability of BMSCs, subsequently, we set the different concentrations of SF (50, 40, 20, 15, 10, 5 and 1 μg/ml) combined with BP (0.75 μg/ml) to incubate with BMSCs for 48 h. Prior to each assay, culture medium in each well was replaced by 100 μl fresh medium containing 10 μl of 5 mg/ml MTT stock solution. After 4 h of labeling cells with MTT, medium was removed and replaced with 100 μl DMSO in each well for 10 min at 37 °C. Samples were mixed and absorbance was set at 540 nm by using an enzyme-linked immunosorbent assay reader [18]. Twelve replicated wells were included in each group, and means were calculated. Finally, when we decided the optimal dosage of SF combined with BP treatment for BMSC, four groups were established which included normal group as control, SF (5 μg/ml) group, BP (0.75 μg/ml) group and SF + BP group.

### Western blot analysis

Western Blot assay was conducted according to our previously described method [17]. The protein samples came from four groups’ BMSCs incubated with different therapeutic agents after 24 h in vitro and cortex of ischemic hemisphere of each group (n = 7) on the 7th day after the operation in vivo, and protein concentration was determined by enhanced BCA protein assay Kit (Beyotime Institute of Biotechnology, Shanghai, China). The samples were electrophoresed on sodium dodecyl sulphate–polyacrylamide gel and electrophoretically transferred to PVDF membranes in Tris–glycine transfer buffer. Then, membranes were blocked with 5 % (w/v) instant non-fat dried milk for 1 h at room temperature, and incubated with rabbit primary antibodies corresponding to β-actin (internal control) (1:1000), VEGF (1:1000), BDNF (1:100), AKT (1:1000), p-AKT (1:2000), mTOR (1:1000) and p-mTOR (1:1000) at 4 °C overnight. The membranes were subsequently washed with TBST [50 mM Tris–HCl (pH 7.4), 150 mM NaCl, 0.05 % Tween 20] and then incubated with secondary goat anti-rabbit IgG (H + L) (1:5000; LI-COR, USA) for 1 h at room temperature. The bands were visualized with Odyssey Infrared Imaging System (LI-COR, USA). The expressions of VEGF and BDNF were normalized against that of β-actin, phosphorylated levels of AKT and mTOR were analyzed by total levels of corresponding protein. The assay was replicated for three independent times.

### Statistical analysis

Statistical calculations were performed with Statistical Product and Service Solutions (SPSS) (version 17.0, Chicago, IL, USA) by one-way analysis of variance followed by least significant difference test for multiple comparisons. Data were expressed as mean ± standard deviation (SD). Differences were considered to be statistically significant at *p* < 0.05.

## Results

### Characterization of cultured BMSCs

**Fig. 1:**
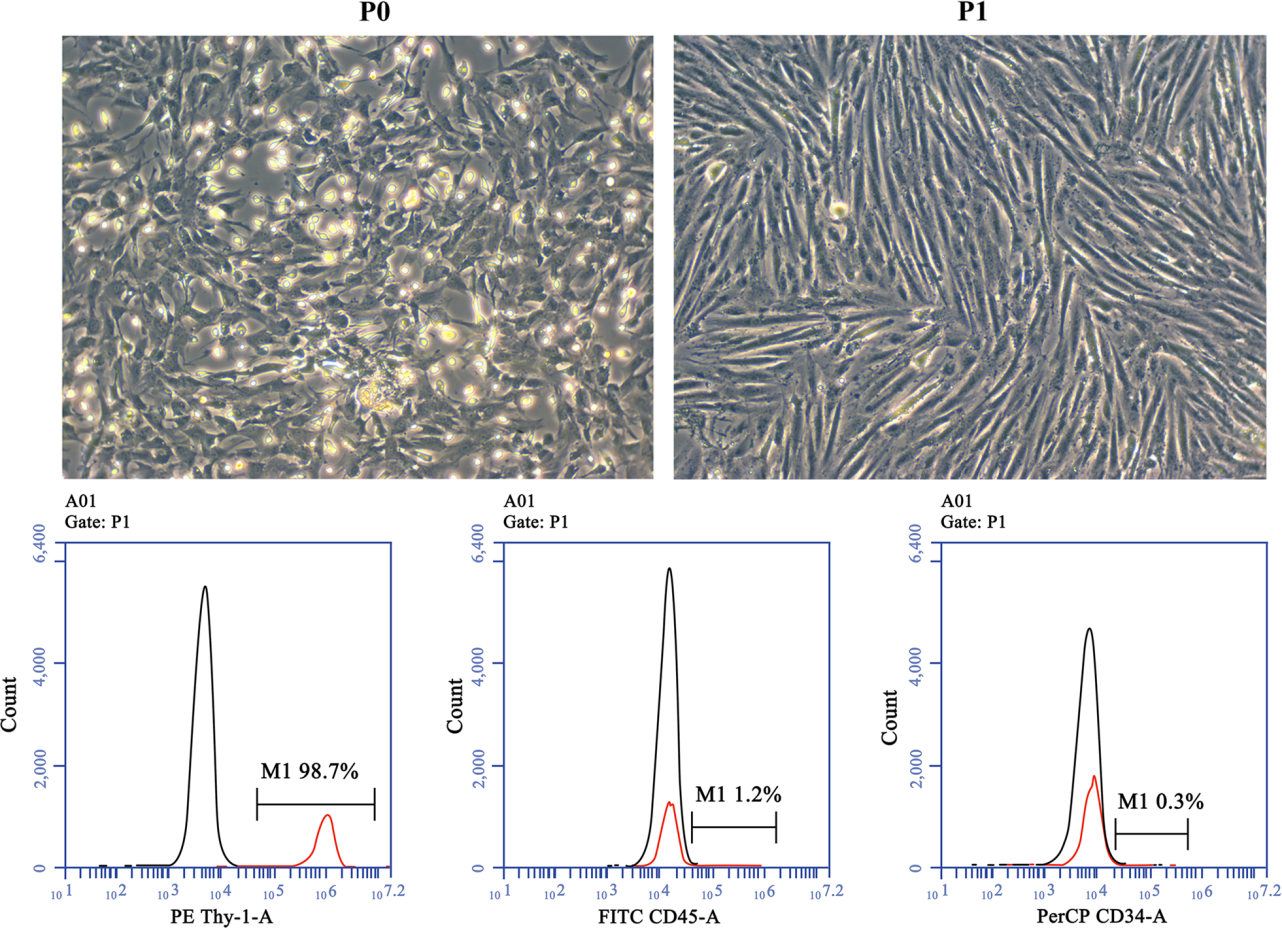
BMSC morphology and phenotype identification by flow cytometry. After the initial seeding, the morphology of adherent cells seemed to be fibroblastic-like mononuclear cells and took a logarithm growth appearance; from passage 1, the cells became uniform and grow in whirlpool, radial, or parallel patterns (Magnification, ×100). BMSCs from passage 3 were incubated with fluorescence-conjugated antibodies including CD90-PE, CD45-PerCP, CD34-PerCP and PBS. The positive expression of CD90 was 98.7 %, and negative identifications of CD45 and CD34 were 1.2 and 0.3 %, respectively

As shown in Fig. 1, from the primary cultured BMSCs to passage 1, the morphology of adherent cells seemed to be fibroblastic-like mononuclear cells and took a logarithm growth appearance. In the present study, the cells at passage 3 were used to determine the purity by flow cytometry. Report demonstrated that CD90 (Thymocyte antigen 1, Thy-1) is a BMSC surface marker [21], while CD45 and CD34 are hematopoietic cell markers [22]. The respective percentage for expression of CD45 and CD34 was 1.2 and 0.3 %, whereas CD90 positive rate was as high as 98.7 %. This result showed that the derived BMSCs was with high-purity and could be applied for next study.

### SF and BP combined with BMSCs decreased infarction zone after stroke

**Fig. 2:**
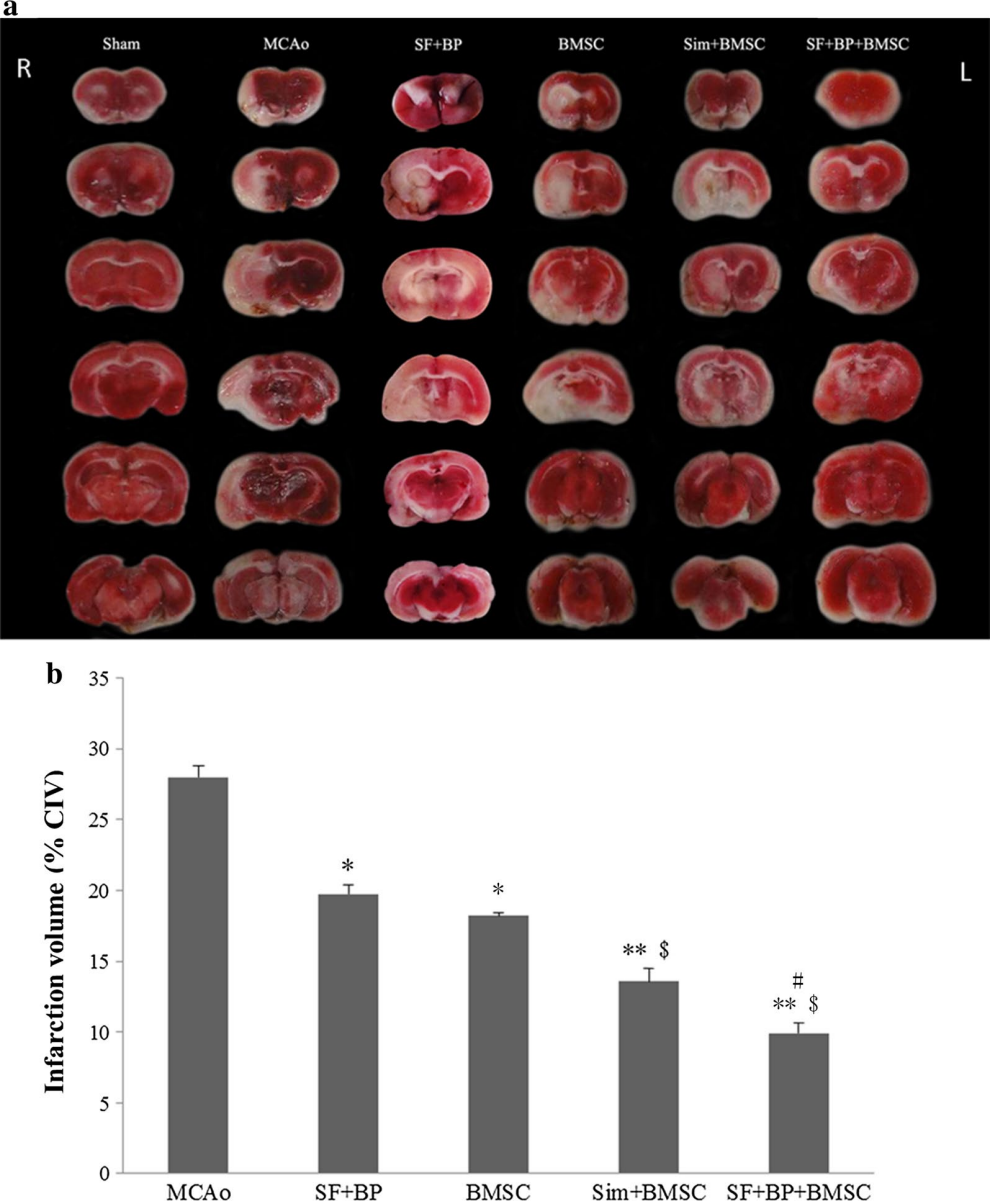
Infarction volume analysis on the 7th day after ischemic stroke. TTC staining of brain slices in Sham, MCAo, BMSC, SF + BP, Sim + BMSC and SF + BP + BMSC groups were presented, and infarction volume in each group was analyzed. Data are presented as mean ± SD. *p < 0.05, **p < 0.01, vs MCAo group, ^#^ *p* < 0.05, vs BMSC group, ^$^ *p* < 0.05, vs SF + BP group

To directly observe the brain infarction zone in each group, TTC staining was used. As shown in Fig. 2, On the 7th day after operation, the infarct volumes in SF + BP + BMSC group (9.93 ± 0.08 %), Sim + BMSC (13.56 ± 0.09 %), BMSC group (18.22 ± 0.02 %) and SF + BP group (19.72 ± 0.07 %) were significantly smaller than that of MCAo group (27.99 ± 0.08 %) (*p* < 0.01, vs. SF + BP + BMSC and Sim + BMSC group; *p* < 0.05, vs. BMSC group and SF + BP group). Furthermore, the infarct volume in SF + BP + BMSC group was notably reduced in comparison with BMSC and SF + BP groups (*p* < 0.05), but there was no statistical difference between the infarction areas in SF + BP + BMSC and Sim + BMSC group. No obvious injury area was observed in sham operated group according to the TTC staining. The above results suggested SF and BP combined with BMSC were capable of decreasing infarct volume post-stroke.

### SF and BP combined with BMSC enhanced VEGF and BDNF expressions in the IBZ

**Fig. 3:**
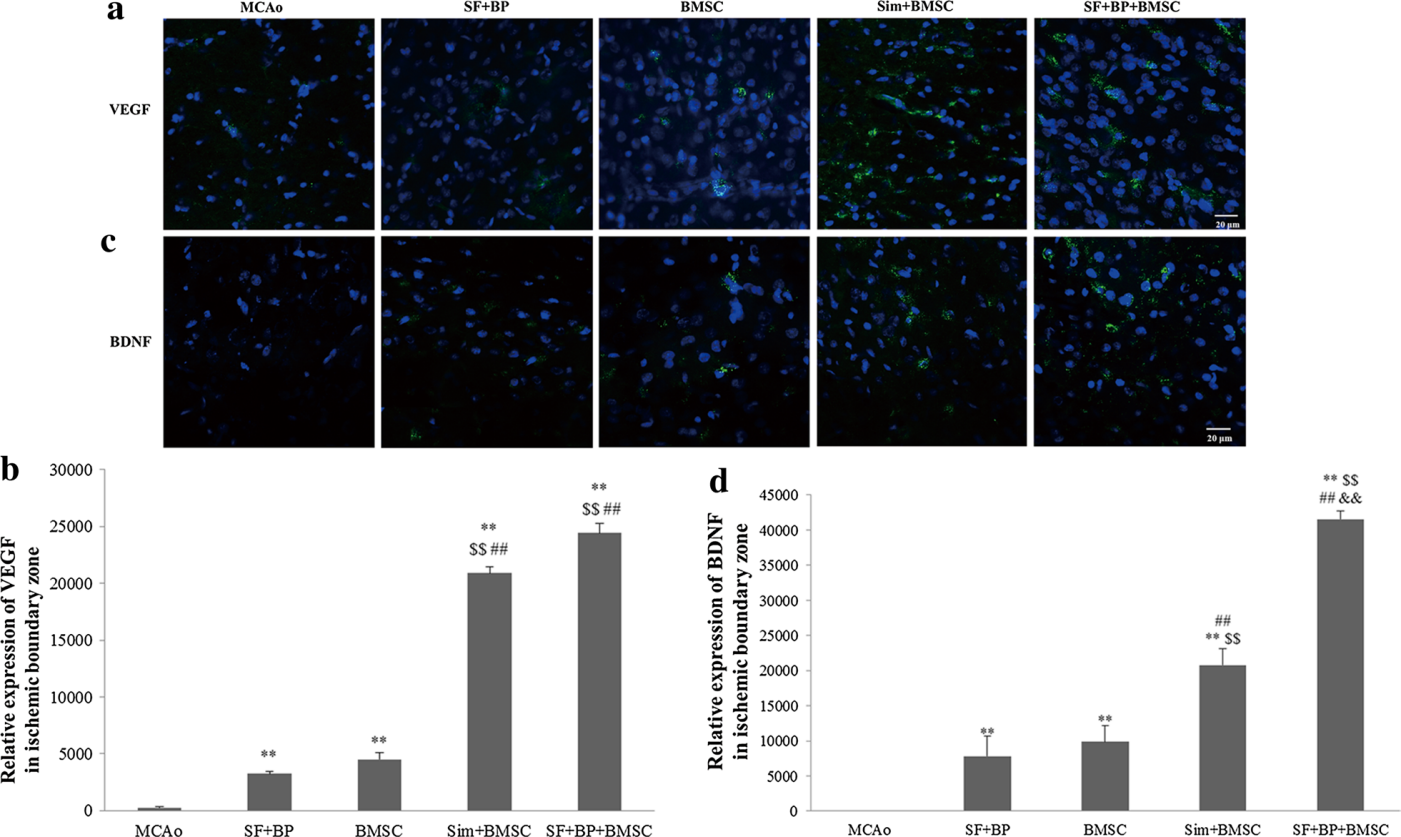
SF and BP combined with BMSC promoted VEGF and BDNF expressions after ischemia. **a**, **c** Immunofluorescence staining images with VEGF and BDNF (*green*) were presented and positive signals were analyzed (**b**, **d**). Data are expressed as mean ± SD. **p < 0.01, compared with MCAo group, ^##^ *p* < 0.01, compared with BMSC group, ^$$^ *p* < 0.01, compared with SF + BP, ^&&^ *p* < 0.01, compared with Sim + BMSC group. The experiment was repeated for 3 times, and representative pictures are shown. *Scale* *bar* 20 μm

As an important angiogenic factor, VEGF plays primary role in facilitating angiogenesis. The quantitative analysis of fluorescent immunostaining image in the IBZ suggested the expressions of VEGF in BMSC alone, Sim + BMSC, SF + BP and SF + BP + BMSC group were more significant than that in MCAo group (*p* < 0.01), and both SF + BP + BMSC and Sim + BMSC treatment could obviously improve VEGF expression compared with BMSC alone treatment and SF + BP group (*p* < 0.01). Although VEGF expression in SF + BP + BMSC group was higher than that in Sim + BMSC group, there was no statistical significance (Fig. 3a, b).

It reported that hydrogel-BDNF advanced immature neurons migration to IBZ and kept them survival for a long time, as well as facilitated axonal sprouting following stroke [23]. Our immunofluorescence assay in Fig. 3c, d suggested the BDNF expression in SF + BP + BMSC group was the most distinguished in all groups (*p* < 0.01, vs. MCAo, SF + BP, BMSC and Sim + BMSC group), and the expression in Sim + BMSC group was notably higher than those in BMSC alone and SF + BP group (*p* < 0.01). It illustrated that SF + BP + BMSC group could dramatically improve BDNF expression in the IBZ.

### SF and BP combined with BMSC increased angiogenesis and neurogenesis in the IBZ

**Fig. 4:**
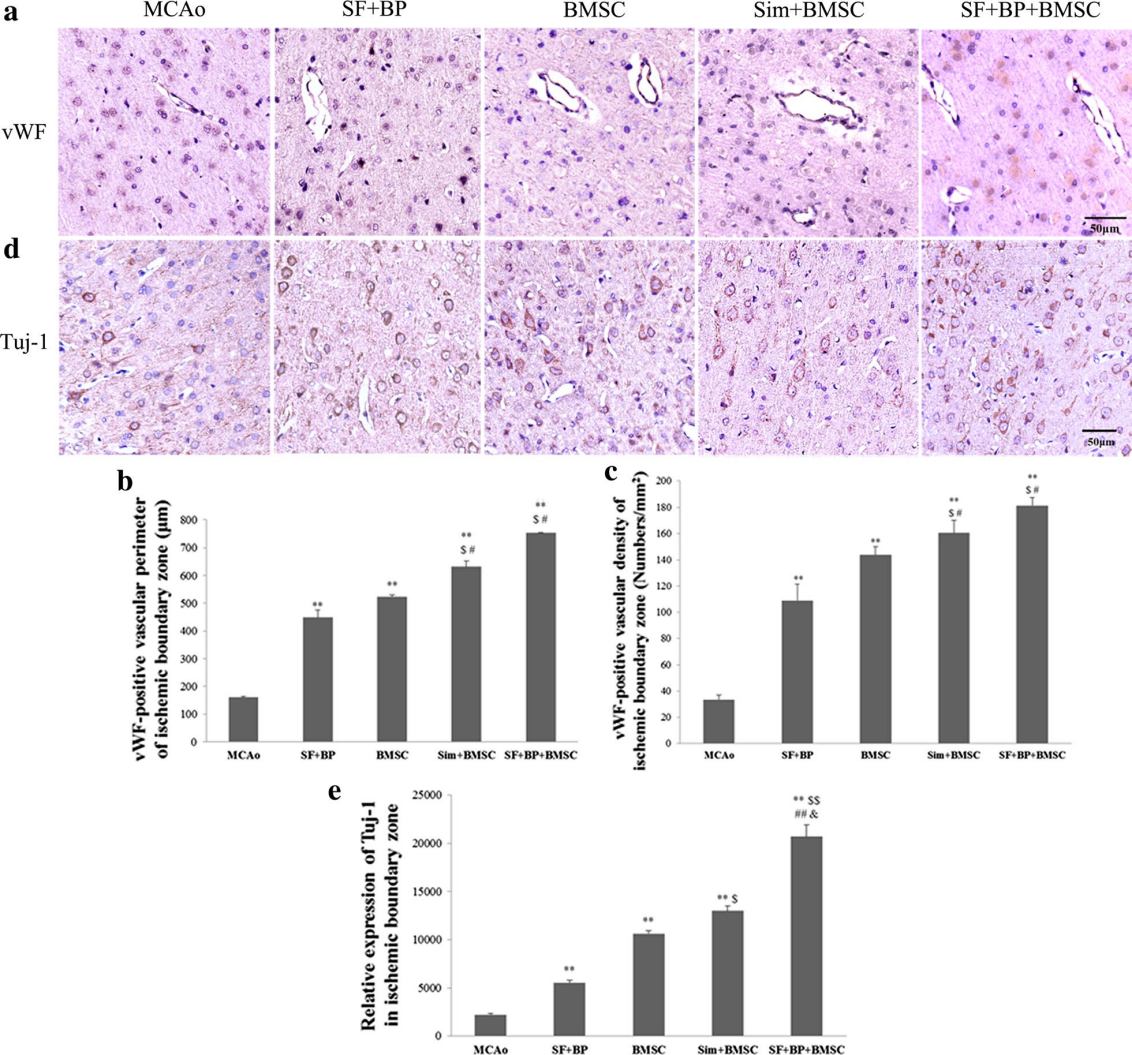
SF and BP combined with BMSC enhanced angiogenesis and neurogenesis after ischemia. **a**, **d** Immunohistochemistry staining images of vWF-vessles and Tuj1 were presented, vWF positive vascular perimeter and density, as well as relative expression of Tuj1 were quantitated (**b**, **c**, **e**). Data are expressed as mean ± SD. **p < 0.01, compared with MCAo group, ^#^ *p* < 0.05, ^##^ *p* < 0.01, compared with BMSC group, ^$^ *p* < 0.05, ^$$^ *p* < 0.01, compared with SF + BP, ^&^ *p* < 0.05, compared with Sim + BMSC group. The experiment was repeated for 3 times, and representative pictures are shown. *Scale* *bar* 50 μm

During the assay of angiogenesis, we observed vWF positive vascular perimeter and the number of positive vWF-vessels in the IBZ by immunohistochemical staining (Fig. 4a, b, c). The results suggested that the two groups of SF + BP + BMSC and Sim + BMSC could more obviously enhance vascular perimeter and density than those in BMSC, SF + BP and MCAo groups (*p* < 0.01, vs. MCAo, *p* < 0.05, vs. SF + BP group and BMSC alone). Although the effects of SF + BP + BMSC group on vWF-positive perimeter and density were higher than Sim + BMSC group, there was no statistical difference between them. It illustrated that SF + BP + BMSC group had the same capable of promoting angiogenesis as Sim + BMSC group in the IBZ post-stroke.

In order to further demonstrate the combination therapeutic effect on neurogenesis, Tuj1 was determined. As a marker of immature neurons, quantitative analysis indicated that SF + BP + BMSC, Sim + BMSC, BMSC and SF + BP groups could obviously elevate Tuj1 expression in comparison with MCAo group (*p* < 0.01), and Tuj1 expressions in Sim + BMSC group was higher than that in BMSC group, but there was no significant difference between the two groups. Additionally, SF + BP + BMSC group could notably promote Tuj-1 expression compared with Sim + BMSC group (*p* < 0.05, Fig. 4d, e). Moreover, we also test doublecortin (DCX, a microtubule-associated protein expressed by neuronal precursor cells and immature neurons) expression in ischemic hemisphere by western blot, it suggested SF (60 mg/kg) + BP (10 mg/kg) + BMSC group significantly improved DCX expression compared with Sim + BMSC group and BMSC alone (*p* < 0.05, see in Additional file 1). The above results demonstrated that SF and BP combined with BMSCs advanced neurogenesis, whose effect was superior to simvastatin plus BMSC administration.

### The definition of optimal dosages of SF and BP treatment for BMSCs in vitro

**Fig. 5:**
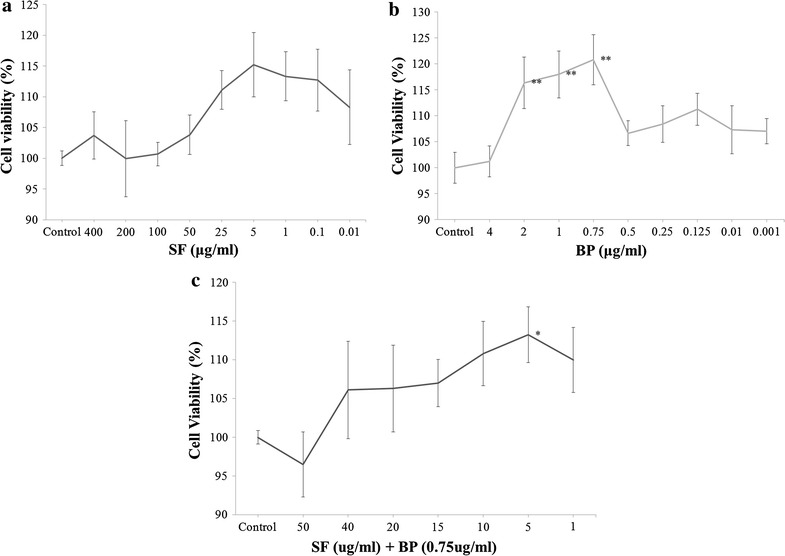
MTT assay for optimal dosages of SF + BP treatment for BMSCs in vitro. BMSCs (1 × 10^5^ cells/ml) were respectively incubated with different concentrations of SF, BP, SF + BP in 96-well plates for 48 h, and the absorbance at 540 nm in every plate was recorded. The viabilities of Cells (**a**, **b** and **c**) were expressed as the percentage of untreated control in the function of different concentrations of SF, BP, SF + BP. We finally selected the SF dosage of 5 μg/ml and the BP dosage of 0.75 μg/ml for next experiment in vitro according to the experimental result

MTT assay was used to identify BMSC viability following different dosages of SF, BP and SF + BP treatment. According to results from the tests, we found that SF at dosage of 1 μg/ml and BP at dosage of 0.75 μg/ml had the most obvious capacities to improve the viability of BMSCs (Fig. 5a, b). In the MTT assay of seven concentrations of SF combined with BP (0.75 μg/ml) for BMSCs, the results indicated that cell survival rates significantly increased at the SF dosage of 5 μg/ml on the 48 h post-treatment (Fig. 5c). Therefore, we finally selected the SF dosage of 5 μg/ml and BP dosage of 0.75 μg/ml as optimal dosages for next experiment in vitro.

### SF and BP administration triggered BMSCs to express VEGF and BDNF

**Fig. 6:**
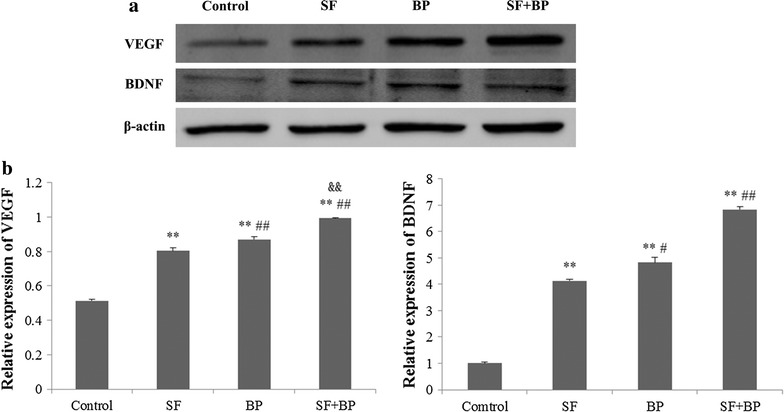
BMSC derived -VEGF and BDNF expressions by western blot assay. Western blot tested the expressions of BMSC derived-VEGF and BDNF in four groups (control, SF, BP and SF + BP group). Representative western blot results for VEGF and BDNF and quantitative analysis at 24 h after treatment. Data are expressed as mean ± SD. **p < 0.01, vs control group, ^#^ *p* < 0.05, ^##^ *p* < 0.01, vs SF group, ^&&^ *p* < 0.01, vs BP group

Western Blot analysis was used to quantify the protein expression levels of VEGF and BDNF derived from BMSCs at 24 h. Figure 6 showed that VEGF and BDNF expressions in SF + BP group were significantly high in comparison with control, SF and BP groups. The above results illustrated that combination of SF and BP might have an additive effect on the improvement of VEGF and BDNF expressions in BMSCs.

### SF and BP combined with BMSC activated AKT/mTOR signaling pathway after cerebral ischemia

**Fig. 7:**
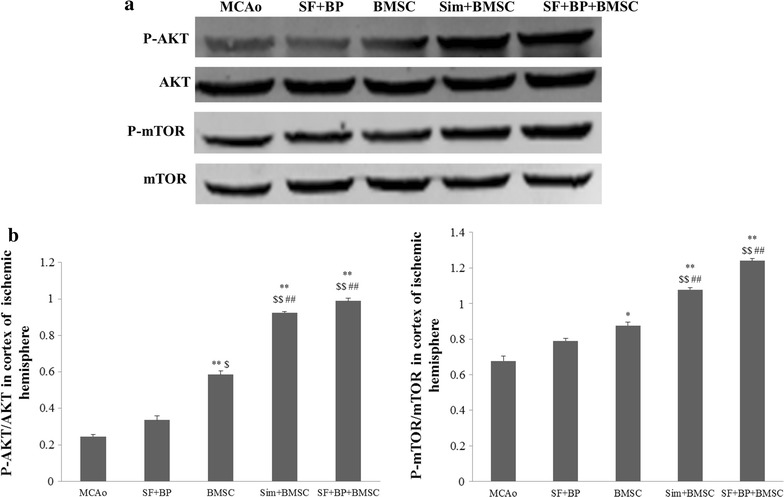
SF and BP combined with BMSC activated AKT/mTOR signaling pathway post-stroke. **a** Representative western blot results for p-Akt, t-Akt, p-mTOR and t-mTOR in the cortex of ischemic hemisphere of five groups were shown; **b** quantitative analysis was presented. Data are expressed as mean ± SD. *p < 0.05, **p < 0.01, compared with MCAo group, ^##^ *p* < 0.01, compared with BMSC group, ^$^ *p* < 0.05, ^$$^ *p* < 0.01, compared with SF + BP group

AKT/mTOR signaling pathway plays a critical role in promoting angiogenesis and neurogenesis. Some research suggested that regulating the activity of mTOR should be developed as a potential therapeutic strategy for ischemic stroke [24]. To test whether our combination treatment of therapeutic agents and BMSC can influence ischemic cortical AKT/mTOR signaling pathway post-stroke, we applied western blot assay. As depicted in Fig. 7, the phosphorylated expressions of AKT in SF + BP + BMSC and Sim + BMSC groups were obviously high compared with those in BMSC, SF + BP and MCAo groups (*p* < 0.01); Meanwhile, quantitative analysis indicated the tendency of mTOR expression in each group was parallel with AKT phosphorylation. It should be noted that SF + BP and BMSC groups had the capabilities of up-regulation of AKT/mTOR expression in comparison with MCAo, but there was no statistics difference between SF + BP and MCAo groups.

## Discussion

**Fig. 8:**
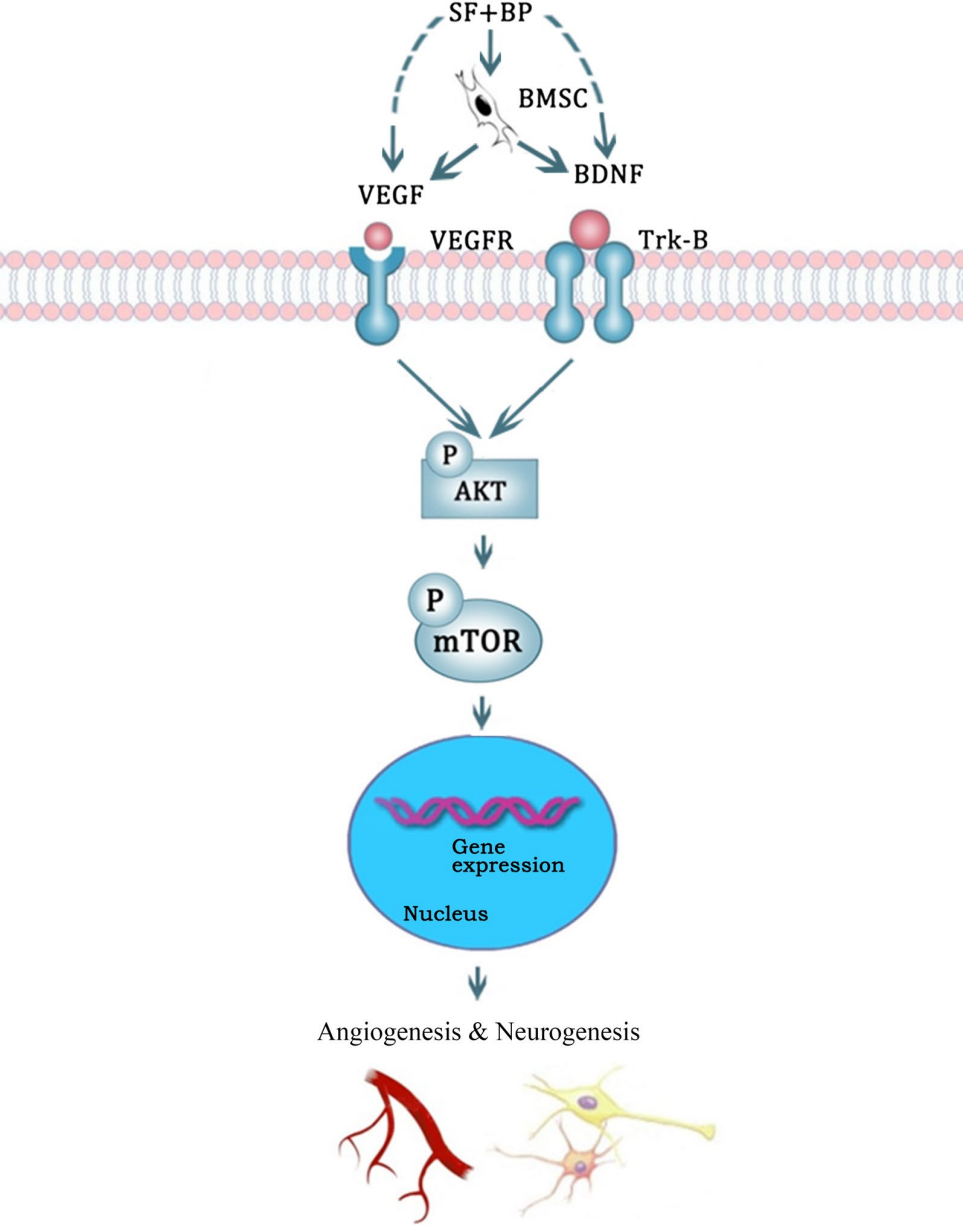
Mechanisms of SF and BP combined with BMSC administration on angiogenesis and neurogenesis following stroke. The effective mechanisms of the combination treatment on angiogenesis and neurogenesis not only include up-regulating VEGF and BDNF-AKT/mTOR signals by SF + BP, as well as BMSC treatments respectively, but also associate with the enhancement of BMSC derived-VEGF and BDNF expressions by SF + BP and subsequently activating AKT/mTOR pathway in cerebral parenchymal cells. Moreover, triggering BMSC paracrine function further improves the additive effects of the combination treatment on angiogenesis and neurogenesis after ischemia

Evidences have showed that grafted BMSCs could improve angiogenesis and neurogenesis which contribute to neurological functional recovery, and the combination treatment of BMSCs and therapeutic drug is further beneficial to the restoration of stroke. However, the debate always existed because the limited number of BMSCs in brain is not parallel with their effects on the amelioration of neurological function. In the present study, we observed that SF and BP combined with BMSC notably improved angiogenesis and neurogenesis in IBZ after ischemic stroke. The synergistic effects were not only related to the enhancement of VEGF and BDNF expressions by SF and BP, as well as BMSC administrations respectively, but also associated with SF and BP advancing BMSC to secrete VEGF and BDNF; meanwhile, the mechanism of the combination treatment might attribute to up-regulating AKT/mTOR signal pathway (Fig. 8).

Amounts of studies have demonstrated that BMSC administration could enhance angiogenesis and neurogenesis; in this sense, BMSC transplantation has been recognized as an effective method for the treatment of stroke [5, 6, 25, 26]. Though the defined mechanism of BMSCs treatment for stroke is still ambiguous, most of experimental results suggested the therapeutic effects was probably relevant to BMSCs paracrine function which would secrete different types of trophic factors resulted in synaptogenesis and neurogenesis, as well as angiogenesis [26, 27]. Previously, Cui and colleagues [10] demonstrated the combination treatment of Simvastatin and BMSC could facilitate BMSCs migration into ischemic area, enhance angiogenesis and ameliorate neurological functional outcome after stroke, and these effects were superior to either Simvastatin or BMSC alone treatment. Our previous study also demonstrated that SF combined with BMSC not only decreased ischemic penumbral injury, but also increased endogenous neurogenesis post-stroke [17], so we speculated the model of combination treatment might be a more potential therapeutic method for ischemic stroke due to synergic functions of supplementary approaches. In the present study, we choose SF and BP combined with BMSC as a dominant treatment model for stroke based on previous results of SF on BMSC and BP on iPSc [15–18]. In order to define optimal dosage ratio of SF and BP in vivo, we tested VEGF, DCX expressions of ischemic hemisphere, as well as neurological functional outcome in different dosages of SF and BP combined with BMSC groups, BMSC alone group and Sim + BMSC group, followed by multiple comparisons in our preliminary experiment, and finally confirmed that the valid dosage ratio of SF and BP was 6:1 (see Additional file 1).

Initial studies have suggested that the occurrences of angiogenesis and neurogenesis are not detached, yet they are coupled processes after stroke [8, 28]. Angiogenesis primarily enhanced oxygen, neurotrophic factors, NSCs migration and nutrient supply to the injured tissue, as well as removed necrotic debris; In addition, cerebral endothelial cells (ECs) from stroke could facilitate NSCs proliferation and differentiation into neurons [7, 28–30]. On the other hand, Teng reported that NSCs from the ischemic subventricular zone could increase capillary tube formation and the coupling of angiogenesis and neurogenesis was substantially regulated by VEGF [28]. As an angiogenic protein, VEGF had exhibited the effects of neuroprotection, angiogenesis and neurogenesis after cerebral ischemia [31]. Furthermore, it had been demonstrated that BMSCs could release VEGF, and simvastatin could further increase BMSCs differentiation into ECs and tube formation through the improvement of VEGF receptor; in this sense, BMSCs treatment will result in angiogenesis post-stroke via mediating VEGF expression [10, 11, 32]. In our present study, SF + BP + BMSC administration dramatically promoted VEGF expression in the IBZ, and the effect was the same as Sim + BMSC group. BDNF played a critical role in promoting the synaptic and axonal plasticity, as well as stimulating human umbilical cord blood-derived mesenchymal stem cells differentiation into neurons after focal ischemia [33, 34]. Additionally, Fouda and colleagues [35] found knockdown BDNF in animal stroke model notably decrease cerebral vascular density, and it suggested BDNF also had important angiogenic function. Our present study found SF + BP + BMSC administration could more distinctly promote BDNF expression in comparison with other four groups; especially, the action was superior to Sim + BMSC group. Moreover, our present study firstly confirmed that the combination of SF and BP could promote BMSCs to synthesize VEGF and BDNF in vitro, and the therapeutic effect was greater than SF or BP alone treatment. So we think the additive effects of the improvement of VEGF and BDNF expressions in SF + BP + BMSC group attribute to three aspects, it includes SF and BP treatment, BMSC treatment respectively, as well as SF plus BP advancing BMSC autocrine action.

To evaluate the capacities of angiogenesis and neurogenesis in different groups, we test vWF-vessel density and vascular perimeter, as well as Tuj-1 expression. The results suggested vWF-vessel density and vascular perimeter had not significant difference between SF + BP + BMSC and Sim + BMSC groups, although the two groups had more distinctly abilities of enhancing angiogenesis in comparison with BMSC and SF + BP groups. As a newborn neuron marker, Tuj-1 is always used to reflect neurogenesis in the study of stroke [36]. Our results showed that Tuj-1 expression in SF + BP + BMSC group was the highest. Though study suggested statin was capable of increasing neurogenesis, synaptic protein and synaptophysin after stroke [9], Simvastatin combined with BMSC in the present study did not obviously promote neurogenesis compared with BMSC treatment. In present study, we did not use VEGF and BDNF siRNA expression vectors or blockage of their receptors to test whether the effects of angiogenesis and neurogenesis were suppressed, but based on previous researches on the actions of VEGF and BDNF post-stroke [28, 31–35), we concluded that the therapeutic effect of SF and BP combined with BMSC on angiogenesis and neurogenesis might relate to the enhancement of VEGF and BDNF expressions in IBZ. According to the analysis of VEGF and BDNF expressions in SF + BP + BMSC group compared with those in Sim + BMSC group, we think the improvement of BDNF by SF an BP combined with BMSC should more contribute to neurogenesis rather than angiogenesis; it is consistent with Jiang and colleagues’ conclusion [37].

In order to further explore the potential mechanism, we detected AKT/mTOR signaling pathway by Western Blot. Previous reports had demonstrated that VEGF interacted with its receptors on the cellular membrane and phosphorylated AKT, subsequently activated mTOR, then up-regulated VEGF expression, and finally improved angiogenesis against cerebral ischemia [38, 39]; recent reports indicated that BDNF could bind with Tropomyosin receptor kinase B receptor and then activate AKT/mTOR signaling pathway which will result in neuroprotective effects, and was beneficial to maintain the endogenous neuronal progenitor pool and regulate new neuron development after stroke [23, 40–43]. Additionally, the activated BDNF/AKT/mTOR signaling pathway also increased the number of cells in the hippocampal dentate gyrus, as well as established a positive feedback loop of BDNF productions following the activation of mTOR by ketamine [44, 45]. Therefore, activated VEGF and BDNF-AKT/mTOR pathway played important roles in angiogenesis and neurogenesis post-stroke. Through quantitative analysis of phosphorylated AKT and mTOR, as well as VEGF and BDNF expressions of each group in vivo and vitro, we thought the effects of the enhancement of VEGF and BDNF-AKT/mTOR cascade in SF + BP + BMSC group not only attributed to SF and BP, BMSC treatments respectively, but also associated with SF and BP advancing BMSC function transposed via non-cell-autonomous signaling. Evidence indicated intravenous injection of BMSCs mainly exist in peripheral organs (lungs, spleen, liver) of MCAo model rather than brain [46], so present consensus has been considered that the amelioration of neurological function of BMSCs is related to their paracrine function, instead of engraftment into the infarction zone. In our study, the up-regulation of BMSC paracrine function embodied that SF and BP triggered BMSCs to synthesize VEGF and BDNF, subsequently AKT/mTOR cascade was activated in cerebral parenchymal cells. It contributed to promoting angiogenesis and neurogenesis in the IBZ, for the reason that we deemed SF and BP should be a “Trigger point”. Interestingly, AKT/mTOR expression in SF + BP + BMSC group was higher than that in Sim + BMSC group, it might be explained why SF + BP + BMSC group could more notably amplify neurogenesis in comparison with Sim + BMSC group. Our present study also suggested that SF and BP combined with BMSC could distinctly reduce infarction volume which perhaps attributed to the improvement of angiogenesis and neurogenesis after ischemia. However, there are limitations in the current study. First, we did not know if there was an additive role between VEGF and BDNF on angiogenesis and/or neurogenesis by the combination treatment. Second, the specific effect and mechanism of SF and BP advancing BMSC paracrine function on which type of cerebral parenchymal cells (e.g. astrocytes, neurons, ECs) should be illustrated in vitro. Third, the effects of SF and BP combined with BMSC on angiogenesis and neurogenesis should not only attribute to the improvement of VEGF and BDNF, as pluripotent cells, BMSCs are sure to have other mechanisms, it needs to be further explored following experiments.

## Conclusion

In summary, our present study firstly demonstrates that SF and BP combined with BMSC can improve angiogenesis and neurogenesis in IBZ and decrease lesion volume post-stroke. The therapeutic effects are associated with the activation of VEGF and BDNF-AKT/mTOR signal pathway. Moreover, SF and BP triggering BMSC paracrine function would more specifically address synergistic efficiency of the combination treatment on ischemic stroke.

## Additional file

10.1186/s12967-016-0979-5 Different dosages of BP combined with SF and BMSC enhanced VEGF and DCX expressions post-stroke. Representative western blot results for VEGF and DCX in ischemic hemisphere of each group (n = 6) were shown and quantitative analysis was presented. It indicated that SF + BP (10 mg/kg) + BMSC group significantly enhanced VEGF and DCX expressions in comparison with other groups. Data are expressed as means ± SD. *p < 0.05, vs. Simvastatin + BMSC group; ^#^p < 0.05, vs. SF + BMSC group. **Figure S2.** Neurological functional outcomes post-surgery. Garcia JH neurological score evaluation was performed at 1 and 7 days in each group (n = 6). Both of SF (60 mg/kg) + BP + BMSC and Simvastatin + BMSC group could dramatically improve neurological functional recovery compared with other four groups on 7th day. Data are expressed as means ± SD. *p < 0.05, vs. BMSC; ^#^p < 0.05, vs. SF (120 mg/kg) + BP + BMSC; ^&^p < 0.05, vs. SF (30 mg/kg) + BP + BMSC.

## Abbreviations

BDNF: brain derived neurotrophic factor
BMSCs: Bone marrow stromal cells
BP: *n*-butylidenephthalide
DMEM/F12: Dulbecco’s modified Eagle medium/F12
ECs: endothelial cells
ELISA: enzyme-linked immunosorbent assay
FA: ferulic acid
FBS: fetal bovine serum
HIF: hypoxic-induced factor
IBZ: ischemic boundary zone
iPSc: induced pluripotent stem cell
MCAo: middle cerebral artery occlusion
NSCs: neural stem cells
PBS: phosphate-buffered saline
SD: Sprague–Dawley
SF: sodium ferulate
TTC: 2,3,5-triphenyltetrazolium chloride
Tuj1: neuronal class III β-tubulin
VEGF: vascular endothelial growth factor
vWF: von Willebrand factor
