# Supplementary material for: Sodium ferulate and n-butylidenephthalate combined with bone marrow stromal cells (BMSCs) improve the therapeutic effects of angiogenesis and neurogenesis after rat focal cerebral ischemia
Source: J Transl Med. 2016 Jul 28;14:223. doi: 10.1186/s12967-016-0979-5 (PMC4963939; doi:10.1186/s12967-016-0979-5)

**Supplementary file**

**The determination of SF and BP therapeutic optimal dosages in vitro**

Our previous study had demonstrated the dosage of SF (60mg/kg) combined with BMSCs (2×10^6^ cells/ml) could significantly improve neurological functional outcome and facilitate neurogenesis in middle cerebral artery occlusion (MCAo) rats [1], so in the present study, we used the dosage as datum line of therapeutic drug. Liu and colleagues [2] found BP (60mg/kg) could notably inhibit neointimal hyperplasia in balloon injured rat carotid artery, therefore, according to the range of dosage, we set three dosages of BP including 40, 20 and 10 mg/kg in the study.

Subsequently, forty-two MCAo rats were randomly divided into seven groups (n = 6 per group), including MCAo group, BMSC group, Simvastatin + BMSC group (positive control, the dosage of simvastatin was determined according to Cui and colleagues [3]), SF + BMSC group, SF +BP (40 mg/kg) + BMSC group, SF +BP (20 mg/kg) + BMSC group and SF +BP (10 mg/kg) + BMSC group; Sham-operated animals (n = 6) were established in order to exclude the influence of the operation process on the therapeutic effect. BMSC suspension solution (2×10^6^ cells/ml), SF (60 mg/kg) and three dosages of BP including 40, 20 and 10 mg/kg were respectively injected into rats in different groups. SF was injected once a day for continuous 7 days, and BP was injected once a day for continuous 3 days because of the enhancement of mortality rate, if BP alone was used for continuous 7 days after stroke.

In order to identify the effects of three dosages of BP combined with SF and BMSCs on angiogenesis and neurogenesis post-stroke, we tested vascular endothelial growth factor (VEGF) and Doublecortin (DCX) expressions in each group on the 7th day post-stroke by Western Blot assay. According to our previously described method, the protein samples incubated with rabbit primary antibodies corresponding to β-actin (internal control) (1:1000), VEGF (1:1000; Abcam, Cambridge, UK) and DCX (1:300; Abcam, Cambridge, UK). The expressions of VEGF and DCX were normalized against that of β-actin. The assay was duplicated for three independent times.

Our result indicated VEGF expressions in SF +BP (20 mg/kg) + BMSC group and SF +BP (10 mg/kg) + BMSC group were dramatically higher than those in Simvastatin + BMSC group and SF + BMSC group (*p*<0.05), and there was no statistical significance between SF +BP (20 mg/kg) + BMSC group and SF +BP (10 mg/kg) + BMSC group. Meanwhile, DCX expressions in the eight groups had the same tendency as the VEGF expressions (Figure S1). It indicated that SF +BP (10 mg/kg) + BMSC group might be the optimal dose combination.

Though the BP dosage of“10 mg/kg ” seemed to significantly improve VEGF and DCX expressions, we did not clearly know if different dosages of SF influenced therapeutic effects. For the reason that we set three dosages of SF including 120, 60 and 30 mg/kg, and thirty-six rats were divided into six groups (n = 6 per group) including MCAo group, BMSC group, Simvastatin + BMSC group, SF (120 mg/kg) + BP + BMSC group, SF (60 mg/kg) + BP + BMSC group and SF (30 mg/kg) + BP + BMSC group. We observed the effects of different SF dosages combined with BP (10 mg/kg) and BMSCs on amelioration of neurological function.

We evaluated neurological functional outcome by Garcia JH test [4], and found Simvastatin + BMSC group could notably increase neurological functional scores compared with other five groups on the first day after ischemia, at the end of therapeutic procedure (Day 7) we found both of SF (60 mg/kg) + BP +BMSC and Simvastatin + BMSC group could dramatically enhance neurological functional recovery compared with other four groups (*p* < 0.05) (Figure. S2).

From the above results, we eventually determined to set “60 mg/kg” and “10 mg/kg” respectively as SF and BP optimal dosages in the present study.

**Figure Legends**

Figure S1. Different dosages of BP combined with SF and BMSC enhanced VEGF and DCX expressions post-stroke. **p* < 0.05, vs. Simvastatin + BMSC group; #*p* < 0.05, vs. SF+ BMSC group.

Figure S2. Neurological functional outcomes post-surgery. Garcia JH neurological score evaluation was performed at 1and 7 days in each group. **p* < 0.05 , vs. BMSC; #*p* < 0.05, vs. SF (120 mg/kg) + BP + BMSC; &*p* < 0.05, vs. SF(30 mg/kg) + BP + BMSC.

**References**

1. Zhao, Y.; Lai, W.; Xu, Y.; Li, L.; Chen, Z.; Wu, W. Exogenous and endogenous therapeutic effects of combination Sodium Ferulate and bone marrow stromal cells (BMSCs) treatment enhance neurogenesis after rat focal cerebral ischemia. *Metab. Brain Dis* 2013, *28*, 655-666.
2. Liu, W.S,; Lin, P.C.; Chang, L.F.; Harn, H.J.; Shiuan, D.; Chiou, T.W.; Jeng, J.R. Inhibitory effect of n-butylidenephthalide on neointimal hyperplasia in balloon injured rat carotid artery. *Phytother. Res* 2011, *25*, 1494-1502.
3. Cui, X.; Chopp, M.; Zacharek, A.; Roberts, C.; Lu, M.; Savant-Bhonsale, S.; Chen, J. Chemokine, vascular and therapeutic effects of combination Simvastatin and BMSC treatment of stroke. *Neurobiol. Dis* 2009, *36*, 35-41.
4. Garcia, J.H.; Wagner, S.; Liu, K.F.; Hu, X.J. Neurological deficit and extent of neuronal necrosis attributable to middle cerebral artery occlusion in rats. Statistical validation. *Stroke* 1995, *26*, 627-634.


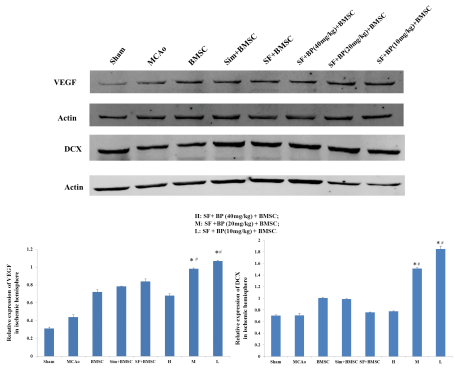

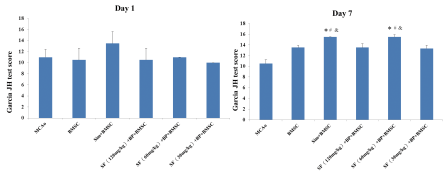

Supplement: Supplementary file 1 — 10.1186/s12967-016-0979-5 Different dosages of BP combined with SF and BMSC enhanced VEGF and DCX expressions post-stroke. Representative western blot results for VEGF and DCX in ischemic hemisphere of each group (n = 6) were shown and quantitative analysis was presented. It indicated that SF + BP (10 mg/kg) + BMSC group significantly enhanced VEGF and DCX expressions in comparison with other groups. Data are expressed as means ± SD. *p < 0.05, vs. Simvastatin + BMSC group; #p < 0.05, vs. SF + BMSC group. Figure S2. Neurological functional outcomes post-surgery. Garcia JH neurological score evaluation was performed at 1 and 7 days in each group (n = 6). Both of SF (60 mg/kg) + BP + BMSC and Simvastatin + BMSC group could dramatically improve neurological functional recovery compared with other four groups on 7th day. Data are expressed as means ± SD. *p < 0.05, vs. BMSC; #p < 0.05, vs. SF (120 mg/kg) + BP + BMSC; &p < 0.05, vs. SF (30 mg/kg) + BP + BMSC. [file 12967_2016_979_MOESM1_ESM.docx]
